# Completeness of Paper-Based Clinical Records as a Dimension of Clinical Record Quality in Selected Public Hospitals in Mpumalanga and Eastern Cape Provinces, South Africa

**DOI:** 10.3390/medsci14040464

**Published:** 2026-08-07

**Authors:** Siyonela Mlonyeni, Ntiyiso Vinny Khosa, Muambangu Jean Paul Milambo, Guillermo Alfredo Pulido-Estrada, Laston Gonah, Thokoe Vincent Makola, Constance Rusike, Wilson Wezile Chitha

**Affiliations:** 1 School of Public Health, Walter Sisulu University, Mthatha 5100, South Africatmakola@wsu.ac.za (T.V.M.);; 2 Walter Sisulu Institute for Clinical Governance and Healthcare Administration, School of Public Health, Walter Sisulu University, Mthatha 5100, South Africa; 3 Biostatistics and Analytics Training Services Unit, School of Medicine and Health Sciences, Walter Sisulu University, Mthatha 5100, South Africa; 4 Department of Human Biology, Faculty of Medicine and Health Sciences, Walter Sisulu University, Mthatha 5117, South Africa; crusike@wsu.ac.za

**Keywords:** clinical documentation, clinical records, documentation completeness, health information systems, data quality, public hospitals, South Africa

## Abstract

**Background:** Clinical documentation is fundamental to quality healthcare delivery, continuity of care, clinical governance, health information management, and medico-legal accountability. However, incomplete clinical documentation remains a persistent challenge in public healthcare settings, and evidence describing documentation completeness at the hospital level in South Africa remains limited. **Aim:** This study aimed to assess the completeness of paper-based clinical records in selected public hospitals in the Mpumalanga and Eastern Cape provinces of South Africa. **Methods:** A quantitative cross-sectional clinical record audit was conducted in four public hospitals located in the Ehlanzeni District of Mpumalanga Province and the OR Tambo District of the Eastern Cape Province. A total of 255 randomly selected patient records from major clinical departments were audited using a structured data extraction instrument. Documentation completeness was assessed across seven domains: patient identification, professional identification, record structure, timing documentation, consent documentation, form completion, and clinical care documentation. Descriptive statistics summarised documentation completeness. One-way analysis of variance (ANOVA) compared mean completeness scores between hospitals, while Fisher’s exact test examined associations between hospitals and overall completeness categories. **Results:** Overall clinical documentation completeness was moderate (mean = 54.5%, SD = 8.1). Timing documentation (96.6%) and clinical care documentation (90.3%) demonstrated the highest completeness, whereas professional identification (37.5%), consent documentation (0.4%), and completion of mandatory clinical forms (1.0%) showed substantial deficiencies. Overall completeness differed significantly between hospitals (F = 14.6, *p* < 0.001), with RFH and Themba Hospital achieving significantly higher mean completeness scores than NMAH and SEH. Most records (69.8%) were classified as having poor completeness, 29.8% demonstrated moderate completeness, and only one record (0.4%) achieved good completeness. **Conclusions:** The findings demonstrate that documentation completeness represents only one dimension of clinical record quality and highlight opportunities to strengthen documentation practices within the participating rural public hospitals. Future research should evaluate interventions that improve documentation completeness alongside other dimensions of clinical record quality.

## 1. Background

The quality of data contained in clinical records is important for delivering efficient healthcare services, ensuring continuity of care, and supporting patient safety and decision-making [1]. Deficiencies in documentation continue to be a problem in many parts of the world, especially in low- and middle-income countries (LMICs) [2]. The WHO states that health information systems are core components of health systems; however, poor data quality, especially in clinical records, continues to negatively affect healthcare delivery [3]. Research shows that 20% to 60% of clinical records are incomplete in LMICs [4,5].

Incomplete clinical documentation remains a persistent challenge across many African health systems. Resource constraints, workforce shortages, and weaknesses in routine health information systems continue to affect the quality and completeness of clinical records, limiting their usefulness for patient care, health service management, and monitoring [5,6]. The problem is even more evident in Sub-Saharan Africa, where health system record-keeping practices are still largely paper-based, error-prone, and easily lost [7]. Various studies conducted in this region indicate that 30% to 70% of clinical records can be incomplete [8,9]. Reported barriers include inadequate documentation training, inconsistent use of standardised documentation tools, and limited supervision of documentation practices [8,9].

The strengthening of health information systems has been given high priority in South Africa through the District Health Information System (DHIS) [10]. Some of the initiatives implemented by the National Department of Health, South Africa, to improve data quality include several efforts; however, issues with missing and inaccurate clinical records continue to exist within the country’s public healthcare system [10]. In a study conducted in public health care settings in South Africa, up to 40% of clinical records have incomplete or missing information [11,12].

Provincial differences in health system resources and service delivery may influence clinical documentation practices [13]. In the Vhembe District, Limpopo Province, staff shortages, high patient volumes, and limited infrastructure create substantial gaps in record-keeping [14]. In the Eastern Cape and Mpumalanga provinces, socioeconomic challenges place additional pressure on health system constraints, particularly in rural and under-resourced facilities [15,16]. Previous studies have reported substantial levels of incomplete clinical documentation in some facilities [17]. These challenges compromise the reliability of health data and hinder effective service delivery and planning. Despite the importance of accurate records, empirical evidence on facility-level data completeness remains limited. Existing research primarily focuses on national or district levels, neglecting hospital-level documentation practices. This study aimed to assess the completeness of clinical records in selected hospitals to inform improvements in data quality and patient care.

## 2. Material and Methods

### 2.1. Study Design, Setting, and Study Population

This study employed a quantitative cross-sectional observational design to assess the completeness of clinical documentation in selected public hospitals in South Africa. The study was reported according to the Strengthening the Reporting of Observational Studies in Epidemiology (STROBE) Statement for cross-sectional studies. A retrospective clinical record audit was conducted using a structured data extraction instrument to evaluate documentation practices within routinely generated patient records. The cross-sectional approach was appropriate because it enabled assessment of existing documentation practices at a defined point in time and allowed comparison of documentation completeness across participating healthcare facilities without altering routine clinical processes.

The study was conducted in four public hospitals located in the Ehlanzeni District of Mpumalanga Province and the OR Tambo District of the Eastern Cape Province, South Africa. The hospitals were purposively selected because they participated in concurrent clinical governance research programmes and represent rural public healthcare environments where health system performance challenges have been documented (Health Systems Trust). Both districts are characterised by rural geography, socioeconomic inequalities, poverty, unemployment, and increased reliance on publicly funded healthcare services [15]. Healthcare facilities within these settings have experienced challenges related to infrastructure limitations, workforce constraints, availability of essential equipment, and operational pressures. However, although these contextual factors may influence documentation practices, the present study did not directly measure staffing levels, workload, infrastructure, or governance processes; therefore, these factors are considered contextual explanations rather than measured determinants of documentation completeness.

The study population comprised patient clinical records maintained within the participating hospitals. Eligible records included completed patient files generated during routine clinical care within selected departments and available within hospital records departments during the study period. Records were included if they originated from participating hospitals, belonged to eligible clinical departments, represented completed patient encounters, and contained sufficient information for assessment using the audit instrument. Records were excluded if they represented active admissions, had been transferred to another facility, were unavailable during the audit period, or were damaged or illegible to the extent that objective assessment was not possible.

### 2.2. Sampling Strategy, Variables, and Outcome Measures

Four hospitals were purposively selected based on their participation in the broader clinical governance programme and their representation of rural public-sector healthcare settings. Within each hospital, simple random sampling was used to select eligible patient records from selected clinical departments, including Maternal and Child Health, Psychiatry, Oncology, Outpatient Department (OPD), and other eligible clinical services included in the parent study framework. Five records were randomly selected from each clinical discipline according to the predefined sampling strategy, resulting in a total sample of 255 patient clinical records across the four hospitals. Random selection of records was undertaken to minimise selection bias and improve the representativeness of audited records within each participating department.

The primary outcome variable was clinical documentation completeness. Completeness was assessed across seven predefined documentation domains: patient identification, professional identification, record structure, timing of documentation, consent documentation, completion of required clinical forms, and clinical care documentation. Individual indicators included demographic information, patient identifiers, diagnosis, treatment documentation, follow-up plans, healthcare professional identification, signatures, professional registration details, dates and times of entries, consent documentation, record organisation, and completion of mandatory clinical forms.

Each documentation indicator was scored dichotomously, with complete documentation assigned a score of 1 and absent, incomplete, illegible, or unidentifiable documentation assigned a score of 0. Indicators not applicable to a specific clinical encounter were excluded from the denominator to ensure that completeness scores reflected documentation requirements relevant to each record. Domain completeness scores were calculated as the percentage of completed applicable indicators within each domain. The overall documentation completeness score was calculated as the mean of the seven domain scores, with each domain contributing equally. Equal weighting was used to provide a standardised assessment of documentation completeness across the entire clinical record rather than to assign differential importance to individual documentation domains, for which no accepted weighting standard exists. Consequently, the overall score should be interpreted as a measure of overall documentation completeness rather than the relative clinical importance of individual domains.

Completeness levels were categorised as poor (<50%), moderate (50–79%), and good (≥80%).

Documentation indicators were assessed only when applicable to the specific clinical encounter. For example, consent documentation and procedure-specific forms were assessed only when the clinical record indicated that the corresponding procedure or intervention required documented consent or completion of a mandatory form. Indicators deemed not applicable were excluded from denominator calculations to ensure that completeness scores reflected documentation requirements relevant to each audited record.

### 2.3. Data Collection Instrument and Clinical Record Sources

Data were collected using a standardised structured clinical record audit instrument developed from an extensive review of international literature on clinical documentation quality, health information systems, patient safety, and clinical governance. The instrument was adapted to the South African public healthcare context through alignment with national clinical record requirements, including documentation principles reflected in the National Core Standards (NCS) of the Office of Health Standards Compliance (OHSC), the Ideal Hospital Framework (IHF), and routine medical record review practices implemented within provincial health services.

The primary source of data was the patients’ clinical record, also referred to as the patient file, medical folder, or Integrated Patient File (IPF), which represents the standard medico-legal documentation system used in South African public hospitals. The audited records included routinely maintained clinical documentation such as patient admission records, demographic information, clinical assessment notes, healthcare professional progress notes, nursing documentation, medication charts, investigation reports, referral documentation, informed consent forms, procedure-related forms, and discharge summaries. These records were selected because they represent the complete clinical documentation pathway used for continuity of care, clinical decision-making, quality improvement, and medico-legal accountability.

The audit instrument assessed seven primary domains of documentation completeness: patient identification, professional identification, record structure, timing of documentation, consent documentation, completion of required clinical forms, and clinical care documentation. Individual indicators included demographic details, patient identifiers, diagnosis, clinical assessment, treatment documentation, follow-up plans, healthcare professional names and signatures, professional registration details, dates and times of entries, informed consent documentation, and completion of mandatory clinical forms.

Each documentation indicator was scored according to predefined operational criteria developed before data collection. Indicators were coded as complete when the required information was present and appropriately documented and incomplete when information was absent, illegible, or insufficient for clinical and medico-legal purposes. Domain completeness scores were calculated by dividing the number of completed indicators by the total number of applicable indicators within each domain and multiplying by 100. The overall documentation completeness score was calculated as the mean percentage across all assessed domains. Completeness categories were defined a priori as poor, moderate, or good according to predetermined score thresholds (provided in Section 2.2, allowing transparent interpretation and reproducibility of findings.

Data extraction was conducted using REDCap (Research Electronic Data Capture), a secure electronic data management platform widely used in health research. REDCap was used to develop standardised electronic case report forms, restrict incomplete data entry, maintain audit trails, and securely store extracted clinical record information. The use of REDCap reduced transcription errors and improved consistency in data management across the participating hospitals.

### 2.4. Validity and Reliability of the Data Collection Instrument

#### 2.4.1. Content and Face Validity

Content validity was strengthened through expert review of published clinical documentation audit tools, health information quality frameworks, and South African clinical governance standards. The audit instrument was mapped against internationally recognised documentation quality indicators and South African healthcare requirements to ensure that relevant domains of clinical record completeness were adequately represented.

The draft instrument was reviewed by experts in clinical governance, health information management, epidemiology, biostatistics, and healthcare quality improvement at Walter Sisulu University. The review focused on relevance, completeness, clarity of definitions, and applicability within rural public hospital environments. Recommendations from the expert review process were incorporated before implementation.

Face validity was assessed through consultation with clinicians, medical record personnel, hospital managers, and researchers experienced in clinical record audits. The instrument was reviewed for readability, feasibility, and suitability for use within routine South African hospital documentation systems.

#### 2.4.2. Pilot Testing and Reliability Assessment

Before the main study, the audit instrument was piloted using clinical records that were excluded from the final analysis. The pilot involved the review of 10 patient clinical records selected from the participating hospitals. The pilot records represented routine South African public hospital documentation systems, including Integrated Patient Files (IPFs), patient folders, clinical notes, nursing records, medication charts, investigation reports, consent forms, and discharge documentation.

The pilot assessment evaluated the feasibility of data collection, clarity of documentation indicators, time required to complete each audit, completeness of electronic data capture fields, and consistency in interpretation between reviewers. Minor modifications were made to improve wording, refine operational definitions, and reduce ambiguity in scoring criteria.

Inter-rater reliability was assessed through independent review of pilot records by two trained researchers using the same audit instrument. Inter-rater agreement between reviewers was calculated using Cohen’s kappa statistic, with interpretation based on established reliability thresholds. Differences identified during independent assessments were reviewed by the research team, and consensus decisions were incorporated into the final audit manual. These procedures improved consistency and minimised measurement variation before commencement of the main data collection phase.

Standard operating procedures were developed for all data collectors, including detailed definitions of each indicator, examples of complete and incomplete documentation, and procedures for resolving uncertain cases. Regular supervision and verification of completed audit forms were undertaken throughout data collection to maintain reliability and quality assurance.

#### 2.4.3. Internal and External Validity

Several measures were implemented to strengthen internal validity. These included the use of a standardised audit instrument, researcher training, predefined scoring criteria, random selection of clinical records, electronic data capture using REDCap, and routine data verification procedures. The retrospective record review design eliminated recall bias because assessments were based on existing documentation rather than self-reported clinical practices.

Although hospitals were purposively selected, inclusion of facilities from two provinces with predominantly rural healthcare contexts improved the transferability of findings to similar resource-constrained public hospital settings in South Africa. However, findings should be interpreted cautiously when applied to urban tertiary hospitals, private healthcare facilities, or settings with more advanced electronic medical record systems.

### 2.5. Ethical Considerations

Ethical approval was obtained from the Human Research and Biosafety Ethics Committee of the Faculty of Health Sciences, Walter Sisulu University (Reference No. 070/2026). Additional research permission was obtained from the Provincial Departments of Health in the Eastern Cape and Mpumalanga provinces before commencement of the study, followed by administrative approval from each participating hospital.

The study involved retrospective review of existing patient records only, and no direct patient contact occurred. Individual informed consent was waived by the ethics committee because the study involved minimal-risk secondary use of routinely collected health information and did not involve collection of identifiable patient information.

Confidentiality and privacy were maintained throughout all stages of the research process. No patient names, identity numbers, hospital numbers, or other personal identifiers were recorded on data collection forms. Each audited record was assigned a unique study identification code to ensure anonymity during data management and analysis. Electronic datasets were password-protected and stored on secure institutional computers accessible only to authorised research team members, while hard-copy audit forms were stored in locked cabinets. The study adhered to the ethical principles outlined in the Declaration of Helsinki, including respect for persons, beneficence, non-maleficence, and justice. Findings are presented only in aggregate form to prevent identification of individual patients, healthcare professionals, or participating institutions.

## 3. Results

### 3.1. Overall Completeness Scores

Table 1 presents the completeness scores across the seven documentation domains. Overall clinical record completeness was moderate, with a mean score of 54.5% (SD = 8.1). Among the seven documentation domains, timing documentation (96.6%) and clinical care documentation (90.3%) demonstrated the highest completeness scores, whereas consent documentation (0.4%), form completion (1.0%), and professional identification (37.5%) demonstrated the lowest completeness. Patient identification was generally well documented (88.1%), while record structure showed moderate completeness (67.8%).

### 3.2. Comparison of Overall Completeness Scores Across the Four Selected Hospitals

**Figure 1:**
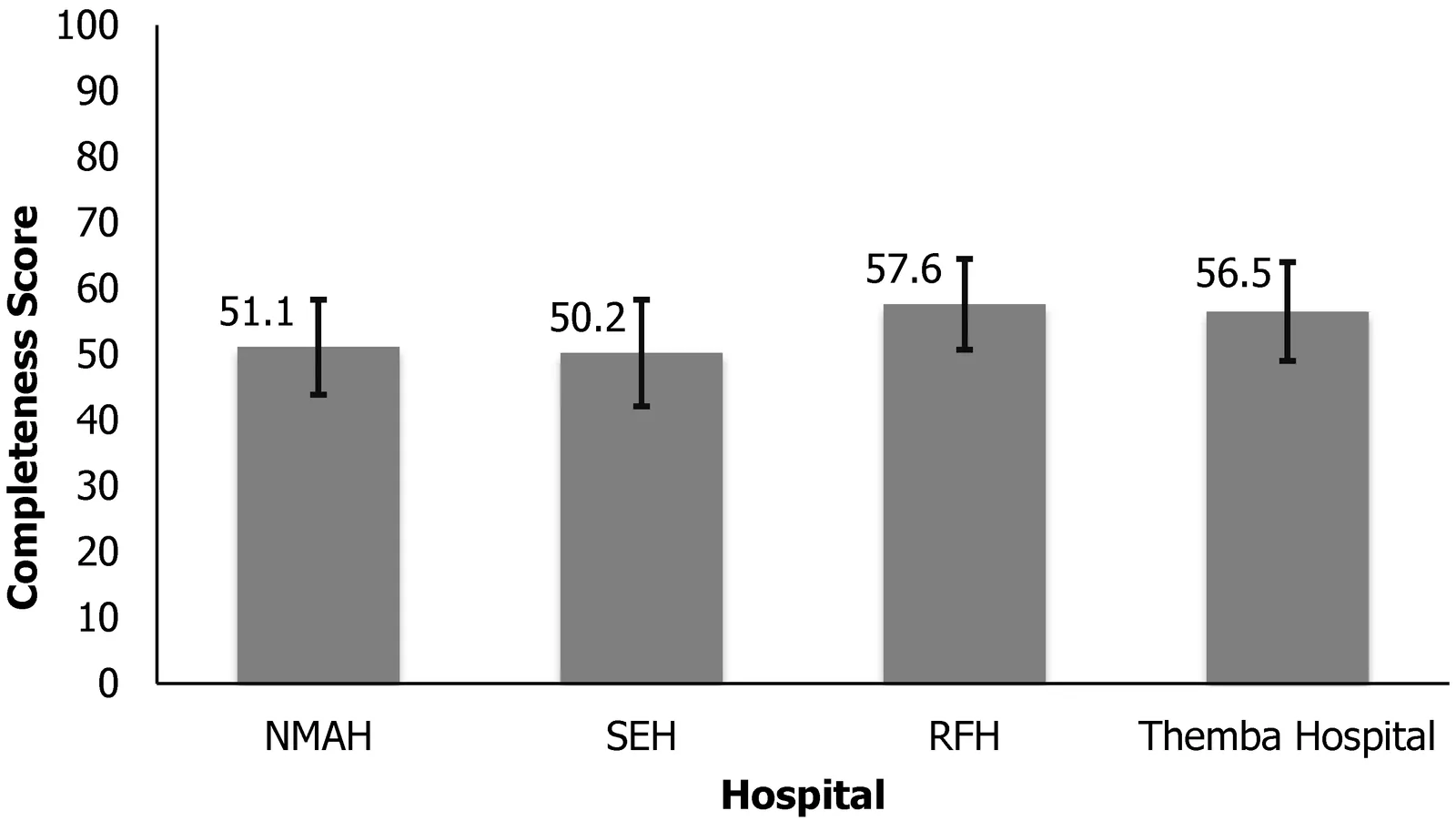
Comparison of overall completeness scores across the four selected hospitals. Bars represent mean overall completeness scores for each hospital, while error bars represent standard deviations. Higher scores indicate greater completeness of clinical records.

Figure 1 illustrates the distribution of overall completeness scores across hospitals. RFH and Themba Hospital achieved higher mean completeness scores than NMAH and SEH; however, none of the hospitals achieved a mean overall completeness score of 60%.

### 3.3. Comparison of Completeness Construct Scores Across Hospitals

Table 2 compares completeness scores across the four hospitals. Significant differences were observed for patient identification (F = 37.2, *p* < 0.001), professional identification (F = 3.4, *p* = 0.018), record structure (F = 29.0, *p* < 0.001), timing documentation (F = 2.7, *p* = 0.047), clinical care documentation (F = 4.0, *p* = 0.008), and overall completeness (F = 14.6, *p* < 0.001). Post hoc analyses indicated that RFH and Themba Hospital achieved significantly higher overall completeness scores than NMAH and SEH, whereas no significant difference was observed between RFH and Themba Hospital or between NMAH and SEH.

Consent documentation and mandatory form completion were not included in between-hospital comparisons because these indicators exhibited substantial zero inflation and limited variability across the audited records.

**Figure 2:**
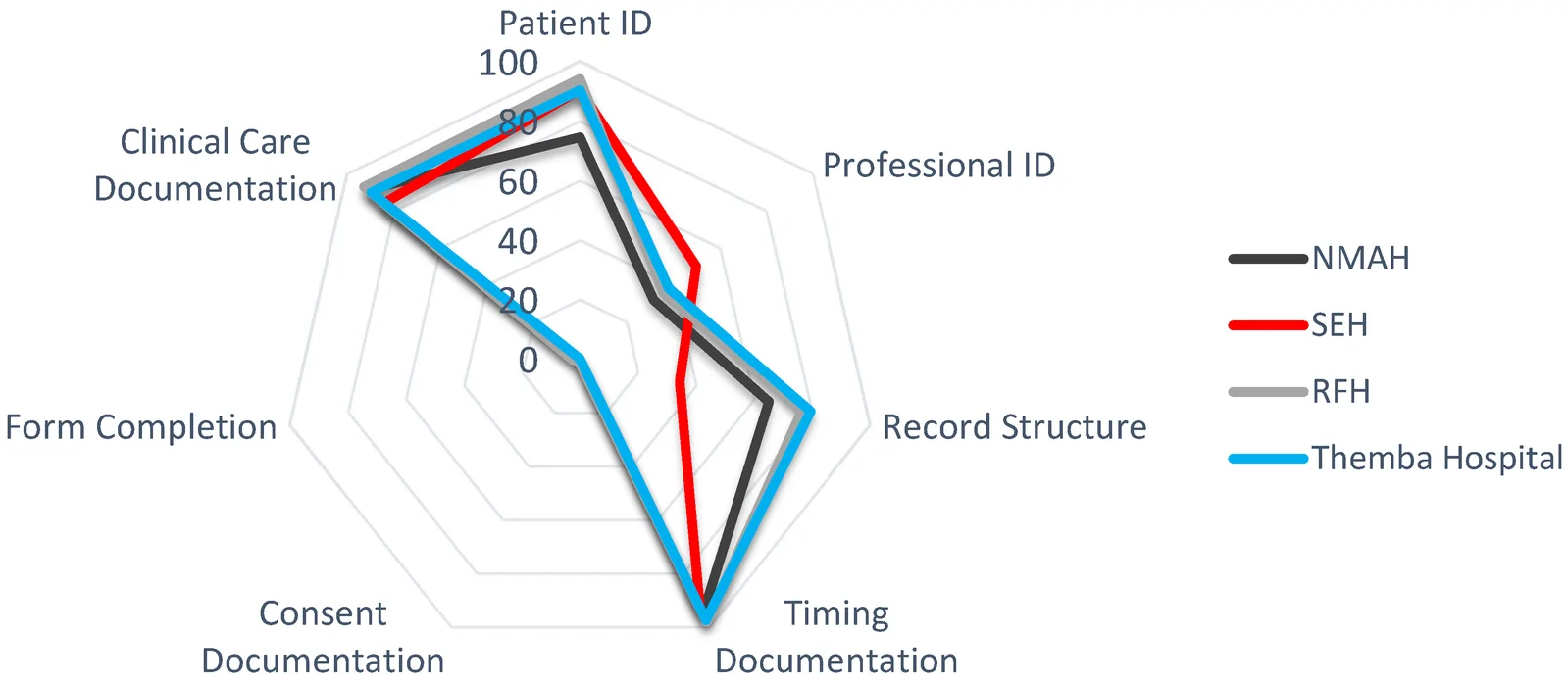
Radar chart comparing completeness construct scores across hospitals.

Figure 2 provides a visual comparison of documentation completeness across hospitals. RFH and Themba Hospital consistently demonstrated higher completeness across most documentation domains, whereas NMAH and SEH performed less favourably. Timing documentation and clinical care documentation consistently achieved the highest completeness scores across hospitals, whereas professional identification, consent documentation, and form completion were the lowest-scoring domains.

Table 3 summarises the completeness of selected documentation indicators. Hospital identification numbers (98.8%), follow-up plans (98.4%), patient/carer involvement (98.4%), and information shared with patients/carers (96.9%) were documented in most records. Conversely, professional authentication indicators demonstrated poor completeness. Identifiable signatures were documented in only 22.8% of records, while staff designation and professional council numbers were absent from all applicable records reviewed.

### 3.4. Association Between Hospital and Overall Completeness Category

Table 4 presents the distribution of overall completeness categories across hospitals. Most clinical records (69.8%) were classified as having poor completeness, while 29.8% demonstrated moderate completeness, and only one record (0.4%) demonstrated good completeness. The distribution of completeness categories differed significantly across hospitals (Fisher’s exact test, *p* < 0.001).

## 4. Discussion

This study assessed clinical documentation completeness as one dimension of clinical record quality across four public hospitals in the Mpumalanga and Eastern Cape provinces of South Africa. Overall documentation completeness was moderate, with documentation directly supporting patient care generally demonstrating substantially higher completeness than documentation supporting professional accountability and medico-legal requirements. Although differences in documentation completeness were observed between participating hospitals, documentation gaps were evident across all facilities, suggesting that incomplete documentation represents a broader challenge within the participating public hospital settings rather than an issue confined to individual institutions.

One of the most important findings of this study was the contrast between relatively complete documentation of patient care activities and poor completion of professional identifiers, consent documentation, and mandatory clinical forms. The observed contrast suggests that documentation supporting immediate clinical decision-making may be prioritised over documentation primarily serving governance, accountability, and medico-legal functions during routine practice. Because organisational factors such as staffing levels, workload, supervision, documentation culture, and resource availability were not measured, the present study cannot determine why this prioritisation occurred. Nevertheless, the consistency of the pattern across multiple hospitals indicates that these documentation gaps are unlikely to represent isolated local practices alone and instead warrant consideration as broader health system challenges requiring further investigation.

The low completeness observed for consent documentation and mandatory clinical forms should be interpreted with caution. Because applicability was determined from information available within the audited clinical records, incomplete documentation may itself have limited identification of situations in which these documentation requirements applied. Consequently, the findings indicate deficiencies in documenting consent and mandatory forms where documentation appeared to be required, rather than demonstrating that these clinical or legal processes were universally omitted.

The observed contrast between documentation supporting direct patient care and documentation supporting governance functions provides an important insight into how clinical documentation is prioritised during routine practice. Previous studies conducted in South Africa and elsewhere in sub-Saharan Africa have similarly reported deficiencies in clinical documentation, particularly regarding professional authentication, consent documentation, and administrative record completion [4,5,8,9,12,18]. However, the contribution of the present study extends beyond confirming these observations. By examining documentation completeness across four hospitals and diverse clinical departments, the findings highlight that documentation gaps were not distributed uniformly across the clinical record. Instead, documentation supporting immediate patient management appeared considerably more complete than documentation supporting governance and medico-legal accountability. This distinction is important because studies evaluating only clinical care documentation may overestimate overall documentation performance while overlooking weaknesses in components essential for accountability, continuity of care, quality assurance, and legal protection.

Although documentation completeness differed between hospitals, these comparisons should be interpreted cautiously. The participating hospitals contributed records from multiple clinical departments with differing documentation requirements, and the study did not adjust for department-specific case-mix or record type. Consequently, observed differences may reflect variation in documentation requirements as well as differences in documentation practice. The findings therefore provide descriptive comparisons rather than evidence of superior documentation performance by particular hospitals. Nevertheless, the absence of any hospital achieving consistently high overall completeness suggests that opportunities for improving documentation practices remain across all participating facilities.

The present findings are also consistent with international evidence demonstrating that documentation directly supporting patient care is often completed more consistently than documentation supporting administrative, legal, and governance functions [19,20]. Studies conducted in settings with mature electronic health record systems have further shown that structured documentation templates, mandatory electronic data fields, automated prompts, and standardised workflows can substantially improve documentation completeness [21,22]. However, these studies also emphasise that improving completeness alone does not necessarily improve the overall quality or usefulness of clinical documentation.

An important methodological implication of the present study is that documentation completeness should not be interpreted as synonymous with overall documentation quality. Completeness represents only one dimension of documentation quality and should be considered alongside other dimensions, including accuracy, consistency, timeliness, usability, and interpretability. A clinical record may therefore appear complete while still containing ambiguous, inconsistent, inaccurate, poorly organised, or clinically uninformative documentation [4,8]. Consequently, high completeness scores should not be interpreted as indicating that documentation is necessarily of high quality or fully supports safe clinical decision-making.

This distinction is particularly important in predominantly paper-based health systems, where clinical information is commonly recorded as unstructured narrative text. Although free-text documentation allows clinicians to capture complex clinical reasoning, heterogeneous narrative records limit the ability to retrieve, aggregate, standardise, and reuse clinical information across health information systems. Recent work has demonstrated that transforming unstructured documentation into standardised clinical concepts through structured terminology and semantic mapping can substantially improve interoperability and secondary use of routinely collected clinical data [23,24]. Consequently, improving documentation completeness alone is unlikely to maximise the value of clinical records unless accompanied by approaches that improve consistency, standardisation, and the computability of clinical information.

The implications of these findings therefore extend beyond documentation completeness alone. Clinical records serve simultaneously as tools for direct patient care, professional communication, organisational accountability, health service monitoring, research, and policy development [4,8]. Persistent deficiencies in professional authentication, consent documentation, and mandatory clinical forms may reduce the traceability, reliability, and medico-legal defensibility of clinical information while also limiting the usefulness of routinely collected data for health system management. Improving documentation should therefore be viewed as strengthening clinical governance and health information systems through improvements in documentation quality rather than simply increasing documentation completeness.

This study contributes to the limited evidence on documentation completeness within South African public hospitals by providing multicentre data from predominantly rural healthcare settings that remain underrepresented in the literature. The findings provide an important baseline against which future documentation improvement initiatives may be evaluated. Future research should move beyond descriptive audits to evaluate interventions that improve both documentation completeness and documentation quality. In particular, studies should investigate the effectiveness of structured documentation models, standardised clinical terminology, semantic interoperability approaches, audit-and-feedback strategies, clinician education, and electronic documentation systems in improving the completeness, consistency, usability, and secondary reuse of routinely collected clinical information within South African public healthcare settings.

## 5. Strengths and Limitations

This study is one of the few multicentre assessments of clinical documentation completeness conducted across public hospitals in the two South African provinces. By including hospitals serving predominantly rural populations, it provides evidence from healthcare settings that remain underrepresented in the clinical documentation and health information quality literature. The use of a standardised audit instrument, predefined scoring criteria, and uniform data collection procedures further enhanced the consistency of the documentation audits.

Several limitations should be considered when interpreting the findings. The cross-sectional design provided only a snapshot of documentation practices and therefore could not establish causal relationships or determine temporal changes in documentation quality. The study also included four purposively selected public hospitals, limiting the generalisability of the findings to urban hospitals, tertiary referral centres, private healthcare facilities, or settings with mature electronic health record systems. Additionally, the participating hospitals contributed records from multiple clinical departments with different documentation requirements and clinical workflows. Because the analyses did not adjust for department, case-mix, or record type, differences observed between hospitals may partly reflect variation in documentation requirements rather than documentation performance alone and should therefore be interpreted cautiously. Again, documentation quality was assessed solely from information contained within patient records; consequently, clinical activities that occurred but were not documented could not be evaluated. Similarly, applicability of consent documentation and mandatory clinical forms was determined from information recorded within the audited files, meaning that incomplete documentation may have itself influenced identification of documentation requirements. Finally, the study focused specifically on documentation completeness as one dimension of clinical record quality. Other important dimensions, including accuracy, consistency, usability, timeliness, and interpretability, were not assessed. In addition, organisational factors such as staffing levels, workload, supervision, documentation culture, and infrastructure were not measured. Future studies incorporating mixed-methods approaches and broader multidimensional assessments of documentation quality would provide a more comprehensive understanding of documentation practices within South African public hospitals.

## 6. Conclusions

Clinical documentation completeness across the four participating public hospitals was moderate overall, although substantial variation was observed across documentation domains. Documentation directly supporting patient identification, timing of clinical entries, and clinical care was generally more complete than documentation related to professional identification, consent, and mandatory clinical forms. Although differences in documentation completeness were observed between hospitals, these findings should be interpreted cautiously because documentation requirements varied across clinical departments and patient pathways. Nevertheless, documentation deficiencies were evident across all participating hospitals, indicating opportunities to strengthen clinical documentation within rural South African public healthcare settings.

The findings further demonstrate that documentation completeness should be interpreted as one dimension of clinical record quality rather than a comprehensive measure of documentation performance. Improving completeness alone is unlikely to maximise the value of clinical records unless accompanied by approaches that strengthen documentation consistency, usability, standardisation, and the secondary reuse of clinical information. Future research should evaluate structured documentation models, standardised documentation approaches, audit-and-feedback interventions, clinician education, and electronic documentation systems to determine their effectiveness in improving documentation completeness alongside other dimensions of clinical record quality in South African public healthcare settings.

## Figures and Tables

**Table 1:** Overall completeness construct scores for the total sample.

Completeness Construct	Mean (SD)	Median (IQR)
Patient identification completeness	88.1 (14.1)	89.0 (22.0)
Professional identification completeness	37.5 (29.4)	33.0 (52.0)
Record structure completeness	67.8 (30.3)	75.0 (50.0)
Timing documentation completeness	96.6 (11.1)	100.0 (0.0)
Consent documentation completeness	0.4 (6.3)	0.0 (0.0)
Form completion completeness	1.0 (9.2)	0.0 (0.0)
Clinical care documentation completeness	90.3 (14.0)	100.0 (17.0)
Overall completeness score	54.5 (8.1)	55.0 (11.0)

Scores are presented as mean (standard deviation) and median (interquartile range), with higher scores indicating greater completeness of clinical records.

**Table 2:** Comparison of completeness construct scores across hospitals.

Completeness Construct	NMAH Mean (SD)	SEH Mean (SD)	RFH Mean (SD)	TH Mean (SD)	F-Statistic	*p*-Value
Patient identification completeness	74.4 (12.4)	90.4 (10.8)	94.1 (8.7)	90.3 (11.2)	37.2	<0.001
Professional identification completeness	31.7 (27.6)	50.0 (32.5)	35.8 (27.8)	38.1 (28.9)	3.4	0.018
Record structure completeness	65.1 (28.4)	34.4 (24.2)	76.7 (25.9)	79.5 (26.3)	29	<0.001
Timing documentation completeness	95.3 (12.6)	93.0 (15.8)	98.3 (7.9)	97.6 (9.8)	2.7	0.047
Clinical care documentation completeness	91.0 (13.2)	84.0 (17.1)	92.7 (11.8)	89.6 (14.9)	4	0.008
Overall completeness score	51.1 (7.2)	50.2 (8.1)	57.6 (6.9)	56.5 (7.5)	14.6	<0.001

Scores are presented as mean (standard deviation), with higher scores indicating greater completeness of clinical records. One-way analysis of variance (ANOVA) was used to compare mean completeness scores across hospitals.

**Table 3:** Selected clinical record documentation indicators across the reviewed records.

Documentation Indicator	Yes *n* (%)	No *n* (%)
Hospital identification number documented	251 (98.8)	3 (1.2)
Patient contact details documented	241 (94.9)	13 (5.1)
Gender recorded	179 (70.2)	76 (29.8)
Signed, identifiable signature present	58 (22.8)	195 (76.8)
Staff designation documented	0 (0.0)	9 (100.0)
Professional council number documented	0 (0.0)	228 (100.0)
Patient name is documented on every page	149 (59.8)	100 (40.2)
Papers securely filed in notes	156 (62.7)	93 (37.3)
Assessments carried out were documented	143 (56.1)	112 (43.9)
Follow-up plan documented	248 (98.4)	4 (1.6)
Patient/carer involvement documented	250 (98.4)	4 (1.6)
Information/leaflets shared and documented	247 (96.9)	8 (3.1)

**Table 4:** Association between hospitals and overall completeness category.

Hospital	Poor *n* (%)	Moderate *n* (%)	Good *n* (%)	Total *n* (%)
NMAH	57 (95.0)	3 (5.0)	0 (0.0)	60 (100.0)
SHE	32 (80.0)	8 (20.0)	0 (0.0)	40 (100.0)
RFH	59 (56.2)	45 (42.9)	1 (1.0)	105 (100.0)
Themba Hospital	30 (60.0)	20 (40.0)	0 (0.0)	50 (100.0)
Total	178 (69.8)	76 (29.8)	1 (0.4)	255 (100.0)

## Data Availability

The datasets generated and/or analysed during the current study are not publicly available because they contain confidential health information obtained from participating hospitals but are available from the corresponding author upon reasonable request and subject to approval from Walter Sisulu University and the relevant Provincial Departments of Health.
